# Towards understanding the chemical environment effect on gold-containing clusters

**DOI:** 10.1186/1758-2946-6-S1-P42

**Published:** 2014-03-11

**Authors:** Doreen Mollenhauer, Nicola Gaston

**Affiliations:** 1 Callaghan Innovation Research Limited, 69 Gracefield Road, 5010 Wellington, New Zealand; 2 MacDiarmid Institute for Advanced Materials and Nanotechnology, Victoria University of Wellington, P. O. 600, 6140 Wellington, New Zealand

## 

Gold clusters and nanoparticles have attracted continuous attention due to interesting and important electronic, catalytic and optical properties [1,2]. As the chemical environment strongly affects the catalytic properties, an understanding of this is essential to be able to control these properties. In order to study the influence of the ligand shell on the catalytic properties we have studied various gold clusters in interaction with different ligands by performing DFT-D3, SCS-MP2 and CCSD(T) calculations [3,4]. The effect of the ligands to the geometric and electronic structure of the gold clusters is analysed in a systematic way [5,6]. Furthermore as bimetallic gold-palladium catalysts have been found to have improved catalytic properties in various reactions in comparison to the monometallic clusters, the influence of the ligand shell is investigated for small systems.
